# Radiomics signature and deep learning signature of intrathrombus and perithrombus for prediction of malignant cerebral edema after acute ischemic stroke: a multicenter CT study

**DOI:** 10.3389/fneur.2025.1650970

**Published:** 2025-09-10

**Authors:** Shuhao Wang, Jingxuan Jiang, Xiaoli Gu, Haiqi Wang, Yangyang Nan, Xiaoyu Xu, Chenqing Wang

**Affiliations:** ^1^Department of Radiology, Guanghua Hospital Affiliated to Shanghai University of Traditional Chinese Medicine, Shanghai, China; ^2^Institute of Diagnostic and Interventional Radiology, Shanghai Sixth People’s Hospital Affiliated to Shanghai Jiao Tong University School of Medicine, Shanghai, China; ^3^Shukun Beijing Technology Co., Ltd., Beijing, China

**Keywords:** radiomics, malignant cerebral edema, acute ischemic stroke, retrospective studies, computed tomography angiography

## Abstract

**Objectives:**

To accurately assess the predictive ability of radiomics and deep learning (DL) features in intrathrombus and perithrombus regions for the risk of malignant cerebral edema (MCE) after acute ischemic stroke (AIS).

**Materials and methods:**

A retrospective study was conducted, enrolling 406 AIS patients who underwent admission CT before endovascular thrombectomy (EVT). Center A patients were randomly divided (7:3) into training/testing sets; Centers B and C formed the external validation cohort. Regions of interest (ROIs) of thrombus and perithrombus were manually delineated and automatically expanded in margin by one pixel. Four hundred twenty-eight radiomic features were extracted from CT images of intrathrombus and perithrombus regions, and 128 DL features were obtained by inputting these images into a VGG16 architecture. Following features fusion, least absolute shrinkage and selection operator (LASSO) regression was employed for dimensionality reduction. Eleven machine learning classifiers were used for model development. Models’ performance was evaluated using Matthews correlation coefficient (MCC) and area under the receiver operating characteristic curve (AUC), with AUC differences tested using DeLong’s method.

**Results:**

MCE occurred in 49 patients (12.1%). In the validation cohort, the logistic regression (LR) models demonstrated discriminative performance with perithrombus (LR-peri: MCC = 0.857, AUC = 0.891), intrathrombus, (LR-intra: MCC = 0.328, AUC = 0.626), and combined (LR-combined: MCC = 0.41, AUC = 0.869) models. The LR-combined model exhibited a significantly superior predictive capacity to that of LR-intra (*p* < 0.05).

**Conclusion:**

Perithrombus features enhance MCE prediction after AIS, enabling optimized medical resource allocation.

**Clinical relevance statement:**

Emphasis is placed on the critical significance of radiomics extracted from the area in and around the thrombus in predicting MCE after AIS, which has far-reaching significance for improving patient prognosis.

## Highlights

- Machine learning models related to thrombosis can effectively predict the occurrence of MCE after AIS.
- The proposed LR-peri radiomics model reached a higher area under the curve (AUC: 0.891, 95% CI: 0.762–1.000).
- Its application provides a beneficial approach for formulating personalized treatment strategies for patients with AIS.

## Introduction

Stroke ranks as the second leading cause of death globally, and MCE is one of the severe complications, with an incidence rate of approximately 10% (1). Cytotoxic edema usually peaks 3 to 4 days after brain injury, but reperfusion of necrotic tissue can cause malignant edema within the first 24 h. Decompressive craniectomy within 48 h improves outcomes and reduces mortality in large-scale infarctions, but unnecessary surgery is highly invasive (2–4). Thus, early and accurate prediction of complications is essential.

CT is the first-line imaging modality for stroke patients on admission and can predict ischemic brain tissue progression. Wen et al. conducted a study on predicting MCE by extracting the CT radiomics features of the middle cerebral artery (MCA) blood supply area from non-contrast computed tomography (NCCT) images of patients with cerebral infarction. Shi et al. demonstrated that the combined Alberta stroke program early CT score and net water uptake (ASPECTS-NWU) could serve as a quantitative predictor of MCE after MCA territory large vessel occlusion, with a moderate positive correlation with the grade of brain edema, indicating that quantitative measurements of ASPECT score, net water uptake, and enhancement ratio based on CT imaging are effective predictive factors for MCE (5, 6). Prior studies have primarily focused on the infarct core, with less research focusing on the impact of the culprit thrombus and surrounding tissue on post-stroke edema. In ischemic stroke, disruption of the blood–brain barrier leads to vasogenic edema, hemorrhagic transformation, and increased mortality. This pathological process is influenced by thrombus characteristics, as research indicates that thrombi with low red blood cell content, high fibrin levels, and elevated extracellular DNA are less likely to achieve first-pass recanalization (FPR). Some studies have also confirmed that the serum inflammatory factor levels and BBB disruption after AIS are associated with the occurrence of vasogenic cerebral edema (7). It is reasonable to assume that the characteristics of the thrombus and surrounding brain tissue can more precisely reflect the inflammatory response and BBB disruption in post-stroke brain tissue (8). Moreover, extracting high-dimensional quantitative radiomic features and deep learning features from medical images to construct machine learning predictive models has its advantages of reducing physician subjective judgment factors and improving accuracy (9–11).

Thrombus and perithrombus radiomic features can predict the origin and prognosis of thrombi. For example, according to our team’s previous research, it was found that (1) thrombus radiomic features could predict the origin and composition of stroke thrombi, and (2) the logistic regression model combining radiomic features from both inside and around the thrombus could effectively assess clinical prognosis after EVT (12–14). However, our previous studies did not involve deep learning features. DL features refer to high-dimensional data representations automatically extracted by multi-layer neural networks, which can effectively capture complex patterns and structures in the data (15). The application of deep learning in the field of stroke covers multiple aspects, from the detection of acute cerebral infarction, lesion segmentation, ASPECTS quantification, to prognostic prediction (16–19). In addition to imaging biomarkers, clinical predictors—including decreased consciousness, nausea or vomiting, and heavy smoking—have been associated with malignant middle cerebral artery (MCA) infarction in hospital-based cohorts (20).

This study aims to fill the gap in the research on predicting MCE using radiomic and deep learning features of the thrombus and its surrounding areas. It deeply explores the possibility of predicting the development of AIS into MCE based on CT thrombus region features, providing strong support for neurosurgeons to formulate personalized treatment strategies.

## Materials and methods

### Patients

**Figure 1:**
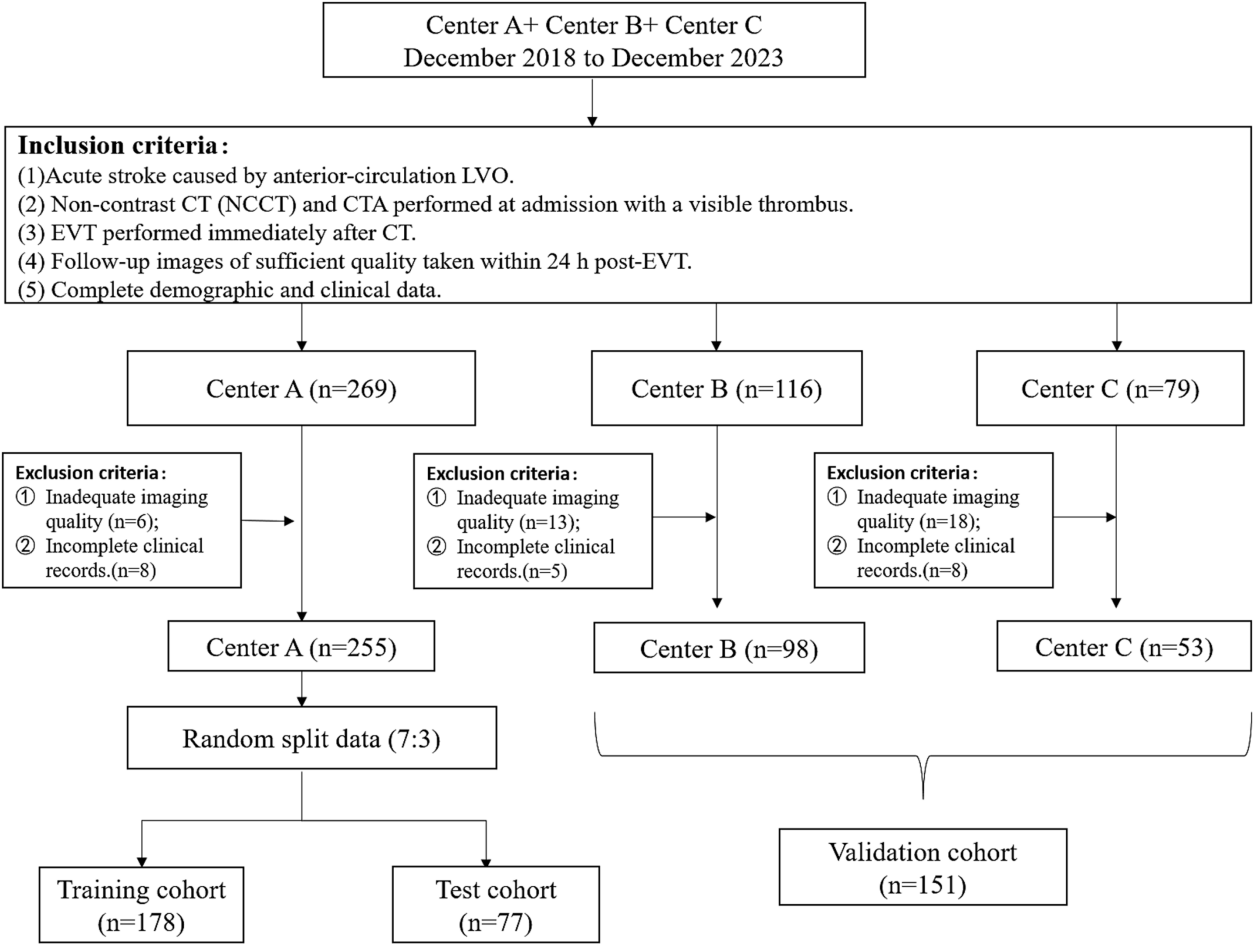
Flow chart of the patient-selection process.

This retrospective study adhered to the 1964 Declaration of Helsinki and its amendments, and was approved by our local ethics committee. All participating centers obtained ethics committee approval with a waiver of written informed consent. We analyzed data from stroke patients admitted to three centers (A: Shanghai Jiao Tong University Affiliated Sixth People’s Hospital, B: Affiliated Hospital of Nantong University, and C: Wuxi Second People’s Hospital) between December 2018 and December 2023. Inclusion criteria: (1) acute stroke due to anterior-circulation large-vessel occlusion (LVO); (2) admission non-contrast computed tomography (NCCT) and computed tomography angiography showing visible thrombus; (3) EVT performed immediately after CT; (4) follow-up images of sufficient quality taken within 24 h post-EVT; (5) complete demographic and clinical data. Exclusion criteria: (1) inadequate imaging clarity due to motion or metal artifacts; (2) incomplete clinical records. Figure 1 shows a flowchart of the patient selection process. The clinical data collected from medical and follow-up records included age, gender, National Institutes of Health Stroke Scale (NIHSS) scores, lesion location, comorbidities (atrial fibrillation, hypertension, hyperlipidemia, diabetes mellitus, and coronary heart disease), and smoking history. MCE was defined as a midline shift of ≥5 millimeters accompanied by signs of local cerebral swelling (21, 22), whose identification was based on follow-up imaging.

### CT scan and thrombosis segmentation

NCCT and CTA examinations were performed using multi-detector CT scanners from three manufacturers: Philips (Brilliance ICT), Toshiba (Aquilion ONE/PRIME), and Siemens (Somatom Sensation). Prior to thrombus segmentation, each CT image underwent an intensity normalization process as described in our previous studies. ROIs for thrombus were outlined using ITK-SNAP software [Version 3.6.0; (ITK-SNAP Home^1^)]. Following segmentation of the intrathrombus areas, perithrombus areas were automatically demarcated by expanding the radius of the initial 1-mm ROIs. To ensure segmentation accuracy, thrombi were segmented by two radiology residents (SHW and XYX) who reached a consensus after consultation, and their work was reviewed by a radiology attending physician (JXJ). Evaluators were blinded to clinical details.

### Feature extraction, selection, and model building

After the identification of regions within and surrounding the thrombus, radiomic features were derived utilizing the PyRadiomics.^2^ The NCCT and CTA images of the area inside and around the thrombus were cropped into fully-covered two-dimensional images. The size of each image was adjusted to 224 × 224 and then input into the VGG16 model. Features were extracted from the final fully connected layer (fc2) of the VGG16 model, which enables the capture of high-level semantic representations of intrathrombus and perithrombus regions. Finally, 107 radiomic features and 32 deep learning features were extracted from the intrathrombus and perithrombus regions on NCCT and CTA images, respectively.

**Figure 2:**
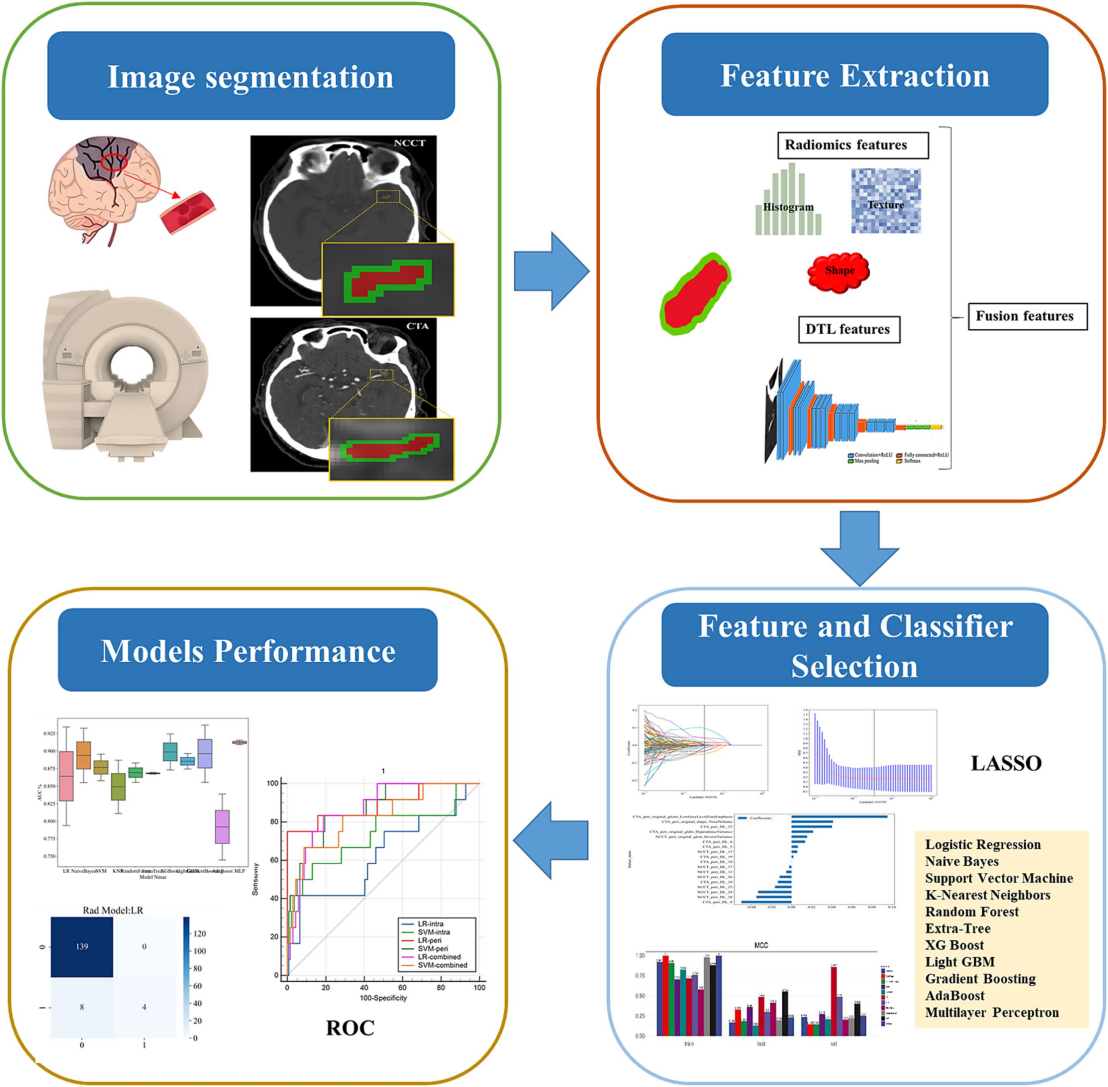
The workflow of this study.

The features with *p* < 0.05 were retained by Mann–Whitney *U* test, and the features with correlation coefficients greater than 0.9 were eliminated according to the Spearman correlation coefficient to achieve dimensionality reduction. After performing feature screening using the LASSO, which included radiomic features and deep learning features, these features were input into machine learning model using 11 different algorithms: Logistic Regression (LR), Naive Bayes (NB), Support Vector Machine (SVM), K-Nearest Neighbors (KNN), Random Forest (RF), Extra-Tree, XG Boost, Light GBM, Gradient Boosting (GB), AdaBoost, and Multilayer Perceptron (MLP). The five-fold cross-validation method was employed to verify the predictive performance of each model. MCC was used to screen optimal algorithm for constructing machine learning models. The performance of these models were evaluated using the ROC curve, along with the AUC, accuracy, precision, specificity, and other metrics. The DeLong test was used to statistically assess the differences in the predictive performance of the machine learning models (intrathrombus, perithrombus, and combined models). The workflow of this study is shown in Figure 2.

### Statistical analysis

Clinical characteristics were analyzed by the *t*-test, Mann–Whitney *U* test, or chi-squared test, as appropriate. The model’s predictive performance for MCE following AIS were evaluated by conducting ROC curve analysis to calculate metrics like the AUC, sensitivity, specificity, positive and negative predictive values, and DeLong’s test was employed to compare ROC curves and assess the model’s clinical utility via decision curve analysis (DCA).

## Results

### Patient characteristics

In this study, 406 AIS patients were, respectively, selected according to the inclusive criteria. Eligible patients from Center A were randomly assigned to the training group (*n* = 178) and the testing group (*n* = 77) at a 7:3 ratio, while those from Centers B and C formed the validation group (*n* = 151). The incidence of MCE after cerebral infarction was 49 cases (12.1%). Table 1 systematically presents the detailed demographic information of the patients in different cohorts classified according to the occurrence of MCE. There was a significant statistical difference in the National Institutes of Health Stroke Scale (NIHSS) score in the training cohort. However, there was no significant statistical difference of NIHSS scores in the testing and validation cohorts. There was also no significant statistical difference among the three groups in terms of other clinical features. For detailed information about the stroke-related characteristics, please refer to the Supplementary material.

**Table 1:** Baseline clinical characteristics of the patients.

Clinical characteristics	Training (MCE^−^)	Training (MCE^+^)	*p*-value	Test (MCE^−^)	Test (MCE^+^)	*p*-value	Validation (MCE^−^)	Validation (MCE^+^)	*p*-value
	(*n* = 148)	(*n* = 30)		(*n* = 70)	(*n* = 7)		(*n* = 139)	(*n* = 12)	
Age	71.72 ± 11.96	69.00 ± 11.88	0.213	62.47 ± 11.74	62.14 ± 17.22	0.946	69.58 ± 11.26	66.42 ± 11.61	0.283
NIHSS	15.08 ± 8.28	10.90 ± 6.26	0.01	13.46 ± 6.24	9.43 ± 4.58	0.101	12.05 ± 5.93	12.92 ± 5.33	0.549
Gender			0.507			1			0.763
Female	67 (45.27)	11 (36.67)		22 (31.43)	2 (28.57)		57 (41.01)	6 (50.00)	
Male	81 (54.73)	19 (63.33)		48 (68.57)	5 (71.43)		82 (58.99)	6 (50.00)	
Atrial fibrillation fibrillation			0.064			0.97			0.539
Absence	89 (60.14)	24 (80.00)		44 (62.86)	5 (71.43)		111 (79.86)	11 (91.67)	
Presence	59 (39.86)	6 (20.00)		26 (37.14)	2 (28.57)		28 (20.14)	1 (8.33)	
Smoke			0.592			0.477			0.4
Absence	118 (79.73)	22 (73.33)		54 (77.14)	4 (57.14)		113 (81.29)	8 (66.67)	
Presence	30 (20.27)	8 (26.67)		16 (22.86)	3 (42.86)		26 (18.71)	4 (33.33)	
Hypertension			0.969			0.785			0.547
Absence	36 (24.32)	8 (26.67)		21 (30.00)	3 (42.86)		51 (36.69)	6 (50.00)	
Presence	112 (75.68)	22 (73.33)		49 (70.00)	4 (57.14)		88 (63.31)	6 (50.00)	
Hyperlipidemia			0.668			0.234			1
Absence	126 (85.14)	24 (80.00)		65 (92.86)	5 (71.43)		138 (99.28)	12 (100.00)	
Presence	22 (14.86)	6 (20.00)		5 (7.14)	2 (28.57)		1 (0.72)	0	
Diabetes			0.85			0.362			0.247
Absence	103 (69.59)	22 (73.33)		56 (80.00)	4 (57.14)		108 (77.70)	7 (58.33)	
Presence	45 (30.41)	8 (26.67)		14 (20.00)	3 (42.86)		31 (22.30)	5 (41.67)	
Coronary disease			1			0.571			1
Absence	119 (80.41)	24 (80.00)		61 (87.14)	5 (71.43)		130 (93.53)	11 (91.67)	
Presence	29 (19.59)	6 (20.00)		9 (12.86)	2 (28.57)		9 (6.47)	1 (8.33)	
Location			0.839			0.104			0.613
MCA	105 (70.95)	22 (73.33)		49 (70.00)	5 (71.43)		90 (64.75)	8 (66.67)	
ICA	31 (20.95)	5 (16.67)		20 (28.57)	1 (14.29)		39 (28.06)	4 (33.33)	
MCA + ICA	12 (8.11)	3 (10.00)		1 (1.43)	1 (14.29)		10 (7.19)	0	

### Feature extraction and selection

**Figure 3:**
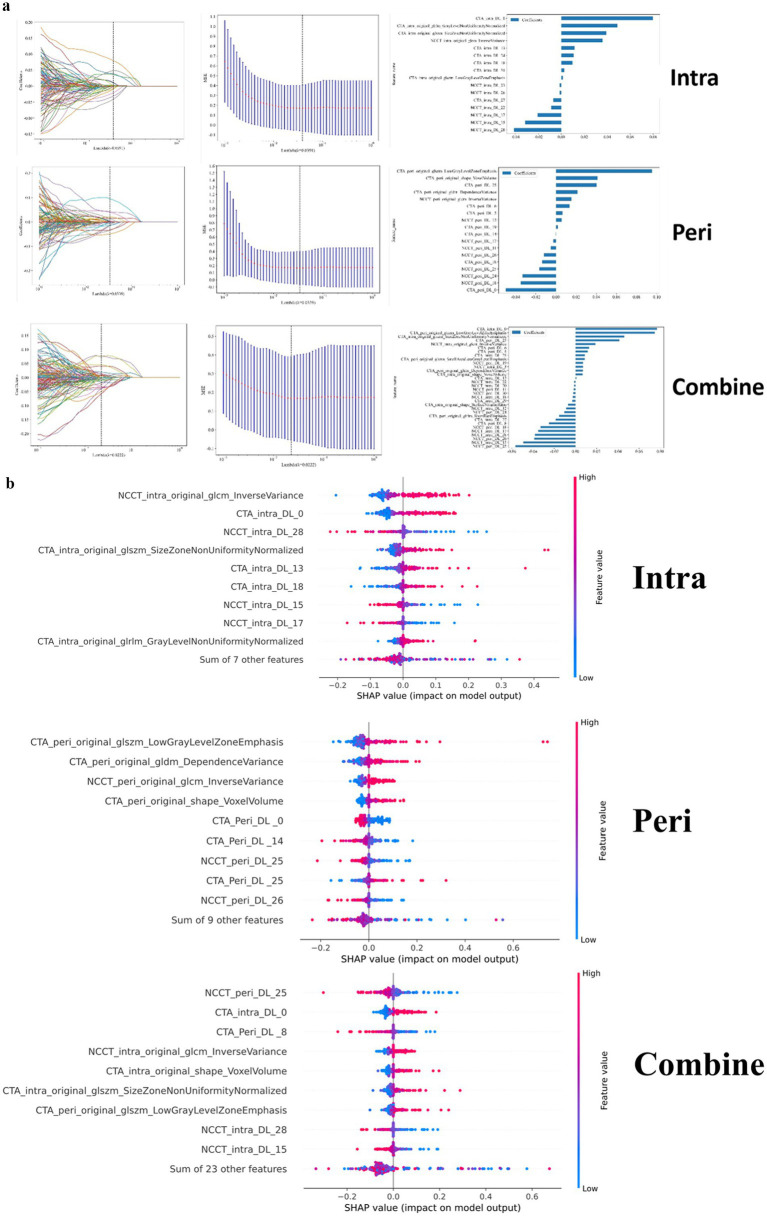
**(a)** Feature selection process. **(b)** SHAP summary dot plot of logistic regression.

After dimensionality reduction, a total of 12 DL features and 4 radiomic features (including 9 CTA features and 7 NCCT features) were used to construct machine learning models based on intrathrombus image features. Regarding the models established based on perithrombus images, they incorporate 14 DL features and 4 radiomic features (including 10 CTA features and 8 NCCT features). Simultaneously, the models developed according to the features of combined are equipped with 24 DL features and 8 radiomics features (including 16 CTA features and 16 NCCT features). Feature selection is shown in Figure 3A. The shapely additive explanation (SHAP) summary dot plot for logistic regression algorithms are shown in Figure 3B.

### Model performance and comparison

**Figure 4:**
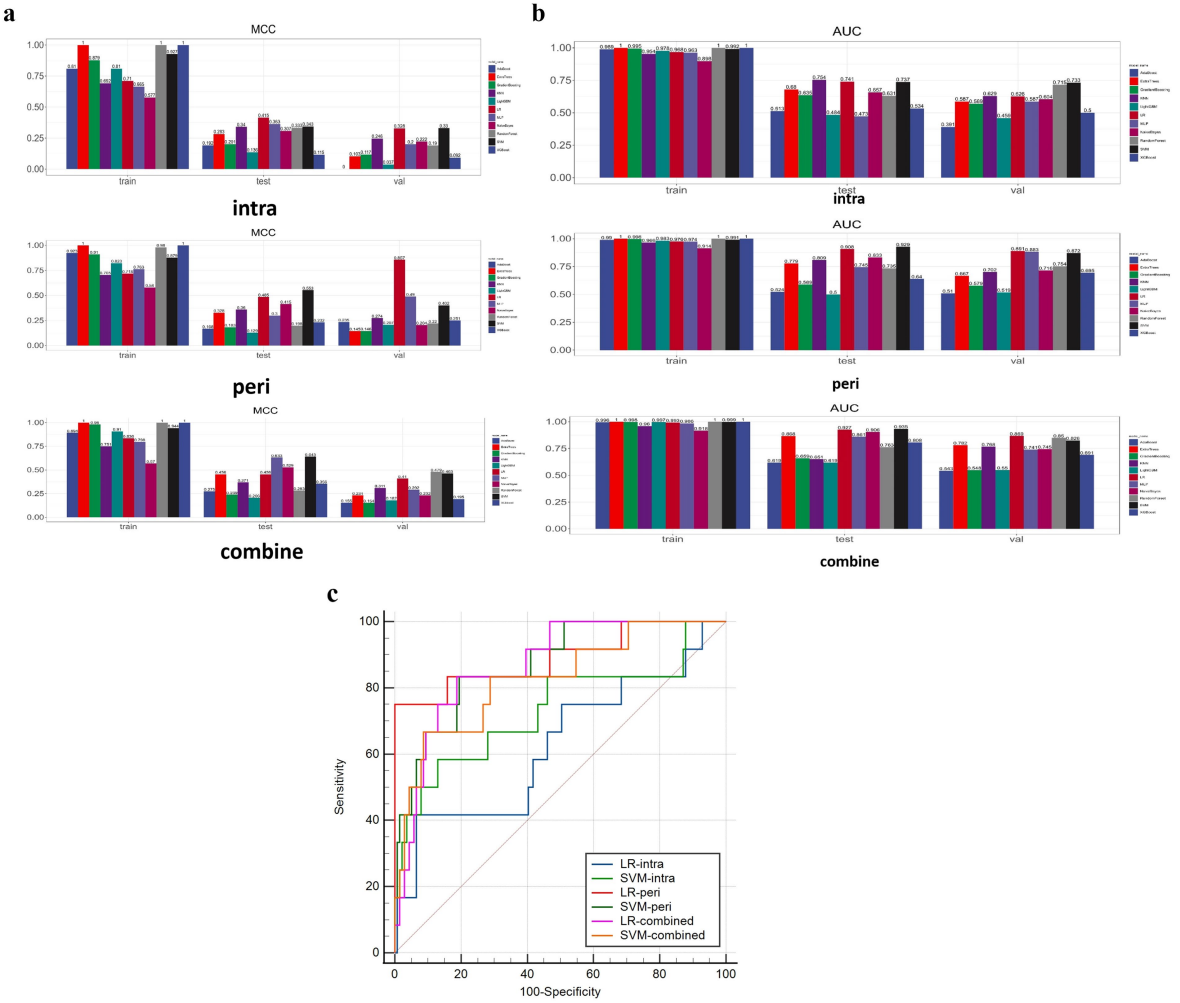
**(a)** Illustrates the MCC results. **(b)** Shows the AUC results. **(c)** Shows the DeLong test results between SVM and LR models of two categories.

The performance of 11 classifiers were evaluated based on MCC values. In the training sets, most classifiers (8 in 11) performed well (MCC >0.7). Figure 4A illustrates the MCC results.

When evaluating the performance of eleven models in the validation set for the perithrombus models, LR performed the best (AUC: 0.891, 95% CI: 0.762–1.000). For the combined models, LR also showed the optimal performance (AUC: 0.869, 95% CI: 0.778–0.9618). For the intrathrombus models, SVM performed well (AUC: 0.733, 95% CI: 0.546–0.921). Figure 4B shows the AUC results. In view of all factors, LR, which performed the best in the predictive task, was selected to construct the prediction models. For detailed information about these models, please refer to Table 2 and the Supplementary material.

**Table 2:** Performance of LR models.

Models	Intra-thrombus			Peri-thrombus			Combined		
	Training	Test	Validation	Training	Test	Validation	Training	Test	Validation
Accuracy	0.876	0.87	0.887	0.888	0.831	0.974	0.938	0.753	0.808
AUC	0.968	0.741	0.626	0.976	0.908	0.891	0.993	0.927	0.869
95% CI	0.9458–0.9907	0.5063–0.9754	0.4325–0.8205	0.9563–0.9950	0.7826–1.0000	0.7615–1.0000	0.9854–1.0000	0.8473–1.0000	0.7776–0.9611
Sensitivity	0.967	0.429	0.333	0.933	0.714	0.667	0.967	0.857	0.75
Specificity	0.858	0.914	0.935	0.878	0.843	1	0.932	0.743	0.813
PPV	0.58	0.333	0.308	0.609	0.312	1	0.744	0.25	0.257
NPV	0.992	0.941	0.942	0.985	0.967	0.972	0.993	0.981	0.974
F1	0.725	0.375	0.32	0.737	0.435	0.8	0.841	0.387	0.383
Threshold	0.196	0.376	0.257	0.182	0.12	0.353	0.2364	0.04271	0.09833
MCC	0.71	0.415	0.328	0.718	0.485	0.857	0.836	0.456	0.41

**Figure 5:**
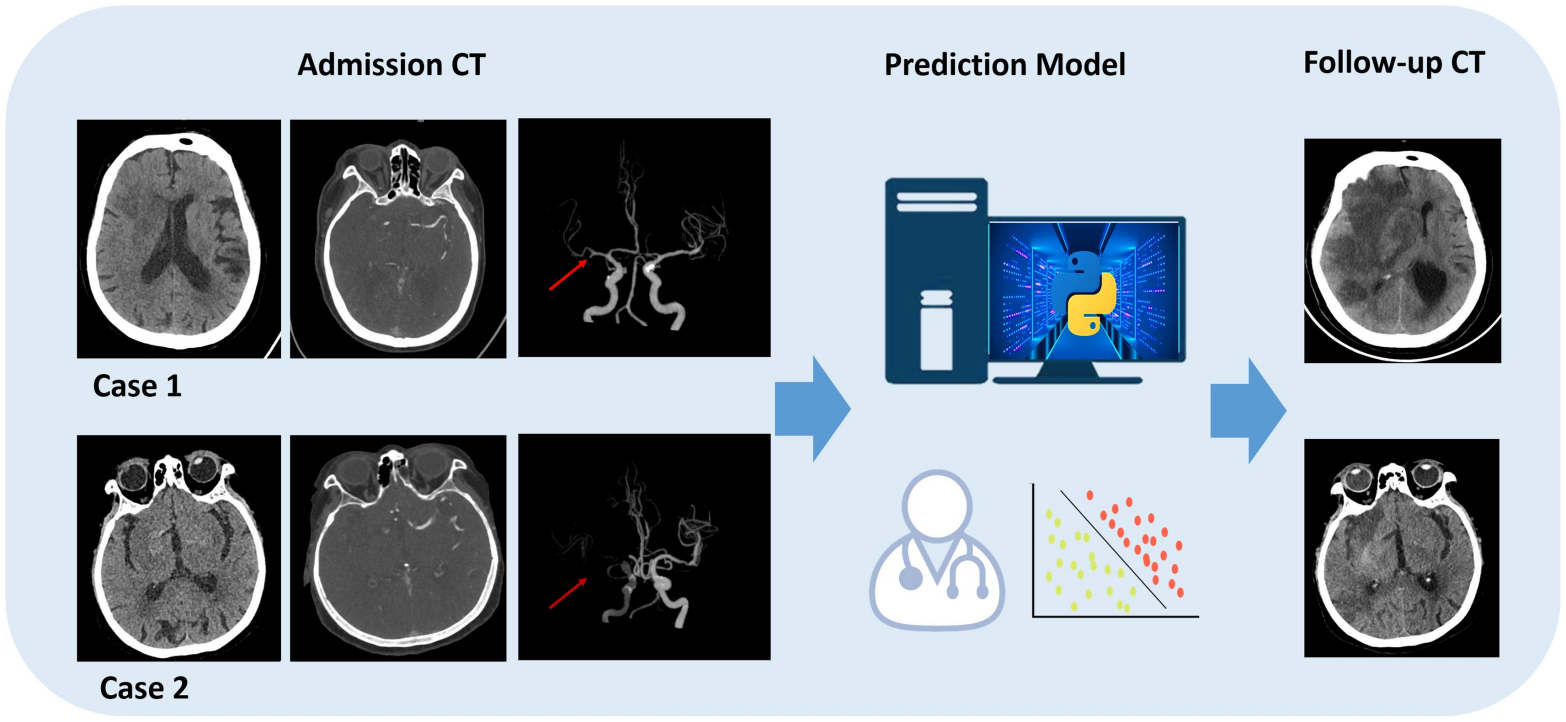
Specific cases are shown. Model prediction in endovascular thrombectomy (EVT) patients. A 45-year-old female predicted and confirmed with post-EVT MCE (case 1), and a 60-year-old male predicted and found without post-EVT MCE (case 2).

In the validation set, the LR-intra model had the lowest AUC value (0.626), showing relatively weak performance in the task. The AUC value of the LR-combine (0.869) was close to that of the LR-peri model. The comparison between LR-intra and LR-combine showed a significant difference (*p* = 0.007), while the comparisons between LR-intra and LR-peri, as well as between the LR-combined and the LR-peri, did not show significant differences (*p* = 0.063 and *p* = 0.788). Figure 4C shows the DeLong test results between SVM and LR models of two categories. During the model validation stage, the DCA indicated that the combined model showed a higher net benefit (0.069), while the perithrombus model had a wider effective threshold probability range (0.08–0.97). Specific cases are shown in Figure 5.

## Discussion

The common cause of AIS is the sudden blockage of the proximal middle cerebral artery or the distal internal carotid artery. The BBB is damaged, leading to excessive water infiltration into brain tissue, with a mortality rate that may be as high as 80% (23, 24). Currently, clinical diagnosis of MCE relies on observing midline shift or brain herniation via CT, which are usually late signs of the disease. The main goal of our research is to develop a model for more early prediction of MCE in AIS patients after EVT. In our study, the perithrombus model exhibited superior predictive capacity in the validation cohort (MCC = 0.857, AUC = 0.891). The model can serve as a tool for early prediction of AIS complications when applied to clinical scenarios, thereby improving the quality of survival of AIS patients after EVT.

Previous developed models mainly extracted image features of the brain infarction area of NCCT images or segmented the MCA supply area of NCCT images. However, it is difficult to accurately delineate the infarcted area on CT images, making it hard to be popularized. Zhang et al. (25) constructed a machine learning model based on the brain infarction area of NCCT images to predict the occurrence of MCE. The model had an AUC of 0.912, showing good predictive ability. Fu et al. (26) evaluated the predictive ability of the IP–NWU value in the middle cerebral artery supply area of NCCT for MCE. The radiomic model had a maximum AUC of 0.96. However, these studies ignored the impact of the responsible blood vessels on the prognosis of brain infarction, and there was little research on deep learning features related to the thrombus area. Additionally, Sarioglu et al. (27) found that thrombus-based radiomic features could effectively predict the first-pass effect (FPE) in patients with AIS. Similar findings were also reported by Xiong et al. (28). When a patient develops AIS, the ischemia and hypoxia of brain tissue caused by the thrombus will damage the BBB, leading to the leakage of macromolecules in the plasma into the brain tissue interstitium due to the increased vascular permeability. The inflammatory response caused by the thrombus will further damage the vascular endothelial cells and exacerbate the vasogenic brain edema. Subsequent reperfusion after revascularization may also aggravate brain injury, thus triggering or exacerbating brain edema. The area surrounding a thrombus includes structures such as vascular wall cells and perivascular fat. It can be seen that the radiomics features around the thrombus can effectively predict MCE and this prediction is interpretable. Li et al. (29) conducted a retrospective analysis of studies related to CT scans before EVT. The research showed that the LR model combining intrathrombus and perithrombus radiomics features was very effective in predicting the prognosis of thrombectomy, with an AUC value as high as 0.87 in the validation cohort. Lu et al. (30) developed a two-stage deep learning model to identify early occult AIS in NCCT. However, we have not seen any relevant research based on the deep learning features of thrombus so far. Inspired by these studies, we constructed a machine learning prediction model for predicting MCE using radiomics and deep learning features extracted from the thrombus and its surrounding areas in NCCT and CTA. The integration of NCCT and CTA radiomics leverages routine clinical imaging to enable rapid MCE risk stratification without additional scans. This approach seamlessly fits into acute stroke workflows, where both modalities are standardly acquired. By extracting predictive intrathrombus and perithrombus features from existing data, our model generates real-time risk scores during the initial interpretation of images. This facilitates early, targeted interventions-such as intensified neuromonitoring for high-risk patients or avoiding unnecessary decompressive surgery in low-risk cases-while optimizing resource allocation in time-critical settings. Future automation could further streamline this process, transforming standard imaging into a prognostic tool for personalized stroke care. Compared with complex models, LR is favored for its statistical simplicity, interpretability, and robust performance in binary classification tasks. Although complex models, like SVM, KNN, RF, Extra Trees, XG Boost, MLP, GB, NB, and AdaBoost, have advantages in handling high-dimensional data, they are prone to overfitting without a large amount of data and careful adjustment. Since the Light GBM model performed poorly in different cohorts of this experiment, which may be related to factors such as the feature distribution of the data, sample size, noise, and the parameter settings of the model, this model should be avoided in future related research. Whereas the consistent performance of LR in the validation cohort confirms its applicability and reliability in clinical diagnosis. Given its effective generalization ability across different datasets, the choice of LR is reasonable.

In the validation cohort, compared with using only the intrathrombus area and the combined area, the radiomics features of the perithrombus area significantly improved the predictive ability for MCE after acute cerebral infarction (AUC: 0.891, 95% CI: 0.761 to 1.000). In validation, the perithrombus model’s high MCC (0.857) versus the combined model (0.41) reflects its perfect specificity in this cohort. While AUC values were comparable (0.891 vs. 0.869), clinicians prioritizing avoidance of overtreatment may prefer the perithrombus model given its extremely high specificity. Conversely, settings emphasizing early case detection may tolerate the combined model’s lower specificity for its higher sensitivity. During model validation, the combined model demonstrated a higher net benefit (0.069) compared to the perithrombus model, which demonstrated a broader effective threshold probability range (0.08–0.97). Clinicians should therefore adopt a dual consideration of net benefit and threshold probability range in clinical decision-making. For instance, integrating the complementary strengths of the combined and perithrombus models may optimize risk stratification compared with the unstable predictive effect of intrathrombus radiomics features, the radiomic features extracted from the perithrombus area play an important role in the prediction model.

In this study, except for the significant statistical difference in the NIHSS index of the training cohort, other clinical variables with or without MCE did not show significant statistical differences in either the training cohort or the validation cohort. Although this study has innovation and important findings, it is still restricted by various factors. The retrospective design inherently limits its causal inference ability, and the relatively insufficient sample size weakens the universality and robustness of the conclusions. Differences in contrast agent selection may interfere with the cross-scenario application of the model. Although the reliability of radiomics feature extraction is high, manual drawing of ROIs may introduce human errors and uncertainties. Future work should explore automated segmentation technologies (e.g., U-Net, nnU-Net) to augment efficiency while preserving diagnostic rigor. The integration of such deep learning tools represents a critical next step for enhancing precision in large-scale applications. Another essential line of future research would be precisely to evaluate the impact of the differences of vascular topography in this topic (31). Anatomical differences in cerebral arterial blood vessels can also predict the prognosis of cerebral infarction. Future studies will investigate data balancing techniques (e.g., SMOTE, class weighting) to further enhance the sensitivity of minority class prediction. Another research direction is to perform spatial multi-omics analysis on thrombectomy specimens to establish associations between imaging biomarkers and drivers of blood–brain barrier disruption (e.g., MMP-9, SUR1-TRPM4). Notwithstanding, challenges including limited multi-center validation, inadequate integration of clinical covariates, and the necessity for advanced hybrid modeling techniques persist. Future investigations should prioritize overcoming these barriers to elevate both research quality and clinical translational value.

## Conclusion

The LR model proposed in this study integrates the NCCT and CTA image features of the perithrombus areas after cerebral infarction through machine learning methods, providing a way to predict MCE. This may help clinicians decide earlier whether to perform decompressive craniectomy or adopt other intensive monitoring measures, ultimately benefiting stroke patients.

## Data Availability

The raw data supporting the conclusions of this article will be made available by the authors, without undue reservation.

## Footnotes

^1^ http://www.itksnap.org/pmwiki/pmwiki.php

^2^ https://pypi.org/project/pyradiomic/
