# Supplementary material for: Radiomics signature and deep learning signature of intrathrombus and perithrombus for prediction of malignant cerebral edema after acute ischemic stroke: a multicenter CT study
Source: Front Neurol. 2025 Sep 10;16:1650970. doi: 10.3389/fneur.2025.1650970 (PMC12457144; doi:10.3389/fneur.2025.1650970)
Supplement: Supplementary file 1 [file Table_1.DOCX]

| Table S1 The performance of eleven machine learning models in the thrombus. | | | | | | | | | | | | | |  |
| --- | --- | --- | --- | --- | --- | --- | --- | --- | --- | --- | --- | --- | --- | --- |
|  |  |  |  |  |  |  |  |  |  |  |  |  |  |  |
| Models | Groups | Accuracy | AUC | 95% CI | Sensitivity | Specificity | PPV | NPV | Precision | Recall | F1 | Threshold | MCC |  |
| LR | Training | 0.876 | 0.968 | 0.9458 - 0.9907 | 0.967 | 0.858 | 0.58 | 0.992 | 0.58 | 0.967 | 0.725 | 0.196 | 0.71 |  |
|  | Test | 0.87 | 0.741 | 0.5063 - 0.9754 | 0.429 | 0.914 | 0.333 | 0.941 | 0.333 | 0.429 | 0.375 | 0.376 | 0.415 |  |
|  | Validation | 0.887 | 0.626 | 0.4325 - 0.8205 | 0.333 | 0.935 | 0.308 | 0.942 | 0.308 | 0.333 | 0.32 | 0.257 | 0.328 |  |
| NaiveBayes | Training | 0.798 | 0.898 | 0.8515 - 0.9440 | 0.933 | 0.77 | 0.452 | 0.983 | 0.452 | 0.933 | 0.609 | 0.054 | 0.577 |  |
|  | Test | 0.857 | 0.657 | 0.4145 - 0.8998 | 0.286 | 0.914 | 0.25 | 0.928 | 0.25 | 0.286 | 0.267 | 0.092 | 0.307 |  |
|  | Validation | 0.795 | 0.604 | 0.4108 - 0.7978 | 0.417 | 0.827 | 0.172 | 0.943 | 0.172 | 0.417 | 0.244 | 0.025 | 0.222 |  |
| SVM | Training | 0.972 | 0.992 | 0.9826 - 1.0000 | 0.967 | 0.973 | 0.879 | 0.993 | 0.879 | 0.967 | 0.921 | 0.216 | 0.927 |  |
|  | Test | 0.779 | 0.737 | 0.4694 - 1.0000 | 0.571 | 0.8 | 0.222 | 0.949 | 0.222 | 0.571 | 0.32 | 0.247 | 0.343 |  |
|  | Validation | 0.841 | 0.733 | 0.5458 - 0.9207 | 0.5 | 0.871 | 0.25 | 0.953 | 0.25 | 0.5 | 0.333 | 0.299 | 0.33 |  |
| KNN | Training | 0.904 | 0.954 | 0.9262 - 0.9815 | 0.5 | 0.986 | 0.882 | 0.907 | 0.882 | 0.5 | 0.638 | 0.4 | 0.692 |  |
|  | Test | 0.922 | 0.754 | 0.5241 - 0.9841 | 0.429 | 0.971 | 0.6 | 0.944 | 0.6 | 0.429 | 0.5 | 0.4 | 0.34 |  |
|  | Validation | 0.894 | 0.629 | 0.4502 - 0.8088 | 0.25 | 0.95 | 0.3 | 0.936 | 0.3 | 0.25 | 0.273 | 0.4 | 0.246 |  |
| RandomForest | Training | 0.994 | 1 | 1.0000 - 1.0000 | 0.967 | 1 | 1 | 0.993 | 1 | 0.967 | 0.983 | 0.5 | 1 |  |
|  | Test | 0.896 | 0.631 | 0.3885 - 0.8728 | 0.143 | 0.971 | 0.333 | 0.919 | 0.333 | 0.143 | 0.2 | 0.5 | 0.333 |  |
|  | Validation | 0.781 | 0.715 | 0.5512 - 0.8793 | 0.5 | 0.806 | 0.182 | 0.949 | 0.182 | 0.5 | 0.267 | 0.2 | 0.19 |  |
| ExtraTrees | Training | 0.831 | 1 | 1.0000 - 1.0000 | 0 | 1 | 0 | 0.831 | 0 | 0 | NaN | 1 | 1 |  |
|  | Test | 0.87 | 0.68 | 0.4419 - 0.9173 | 0.286 | 0.929 | 0.286 | 0.929 | 0.286 | 0.286 | 0.286 | 0.3 | 0.283 |  |
|  | Validation | 0.609 | 0.587 | 0.4237 - 0.7496 | 0.417 | 0.626 | 0.088 | 0.926 | 0.088 | 0.417 | 0.145 | 0.1 | 0.103 |  |
| XGBoost | Training | 0.994 | 1 | 1.0000 - 1.0000 | 0.967 | 1 | 1 | 0.993 | 1 | 0.967 | 0.983 | 0.571 | 1 |  |
|  | Test | 0.403 | 0.534 | 0.3344 - 0.7329 | 0.714 | 0.371 | 0.102 | 0.929 | 0.102 | 0.714 | 0.179 | 0.035 | 0.115 |  |
|  | Validation | 0.517 | 0.5 | 0.3679 - 0.6315 | 0.5 | 0.518 | 0.082 | 0.923 | 0.082 | 0.5 | 0.141 | 0.037 | 0.092 |  |
| LightGBM | Training | 0.927 | 0.978 | 0.9615 - 0.9953 | 0.967 | 0.919 | 0.707 | 0.993 | 0.707 | 0.967 | 0.817 | 0.257 | 0.81 |  |
|  | Test | 0.273 | 0.484 | 0.3071 - 0.6603 | 0.857 | 0.214 | 0.098 | 0.937 | 0.098 | 0.857 | 0.176 | 0.062 | 0.136 |  |
|  | Validation | 0.576 | 0.459 | 0.2983 - 0.6195 | 0.417 | 0.59 | 0.081 | 0.921 | 0.081 | 0.417 | 0.135 | 0.072 | 0.037 |  |
| GradientBoosting | Training | 0.961 | 0.995 | 0.9896 - 1.0000 | 0.967 | 0.959 | 0.829 | 0.993 | 0.829 | 0.967 | 0.892 | 0.226 | 0.879 |  |
|  | Test | 0.623 | 0.635 | 0.3933 - 0.8761 | 0.571 | 0.629 | 0.133 | 0.936 | 0.133 | 0.571 | 0.216 | 0.092 | 0.201 |  |
|  | Validation | 0.444 | 0.569 | 0.4210 - 0.7169 | 0.75 | 0.417 | 0.1 | 0.951 | 0.1 | 0.75 | 0.176 | 0.073 | 0.117 |  |
| AdaBoost | Training | 0.927 | 0.989 | 0.9781 - 0.9996 | 0.967 | 0.919 | 0.707 | 0.993 | 0.707 | 0.967 | 0.817 | 0.478 | 0.81 |  |
|  | Test | 0.61 | 0.513 | 0.2962 - 0.7304 | 0.571 | 0.614 | 0.129 | 0.935 | 0.129 | 0.571 | 0.211 | 0.027 | 0.192 |  |
|  | Validation | 0.073 | 0.391 | 0.2396 - 0.5422 | 0.917 | 0 | 0.073 | 0 | 0.073 | 0.917 | 0.136 | 0.013 | 0 |  |
| MLP | Training | 0.848 | 0.963 | 0.9374 - 0.9883 | 0.967 | 0.824 | 0.527 | 0.992 | 0.527 | 0.967 | 0.682 | 0.175 | 0.665 |  |
|  | Test | 0.909 | 0.473 | 0.2283 - 0.7187 | 0 | 1 | 0 | 0.909 | 0 | 0 | NaN | 0.389 | 0.363 |  |
|  | Validation | 0.775 | 0.587 | 0.3885 - 0.7853 | 0.417 | 0.806 | 0.156 | 0.941 | 0.156 | 0.417 | 0.227 | 0.209 | 0.2 |  |

| Table S2 The performance of eleven machine learning models around the thrombus. | | | | | | | | | | | | | | | | | | | | | | | | | |  |
| --- | --- | --- | --- | --- | --- | --- | --- | --- | --- | --- | --- | --- | --- | --- | --- | --- | --- | --- | --- | --- | --- | --- | --- | --- | --- | --- |
|  |  |  |  |  |  |  |  |  |  |  |  |  |  |  |  |  |  |  |  |  |  |  |  |  |  |  |
| Models | Groups | | Accuracy | AUC | | 95% CI | | Sensitivity | Specificity | | PPV | | NPV | | | Precision | | Recall | | F1 | | Threshold | | MCC | |  |
| LR | Training | | 0.888 | 0.976 | | 0.9563 - 0.9950 | | 0.933 | 0.878 | | 0.609 | | 0.985 | | | 0.609 | | 0.933 | | 0.737 | | 0.182 | | 0.718 | |  |
|  | Test | | 0.831 | 0.908 | | 0.7826 - 1.0000 | | 0.714 | 0.843 | | 0.312 | | 0.967 | | | 0.312 | | 0.714 | | 0.435 | | 0.12 | | 0.485 | |  |
|  | Validation | | 0.974 | 0.891 | | 0.7615 - 1.0000 | | 0.667 | 1 | | 1 | | 0.972 | | | 1 | | 0.667 | | 0.8 | | 0.353 | | 0.857 | |  |
| NaiveBayes | Training | | 0.787 | 0.914 | | 0.8720 - 0.9550 | | 0.967 | 0.75 | | 0.439 | | 0.991 | | | 0.439 | | 0.967 | | 0.604 | | 0.046 | | 0.58 | |  |
|  | Test | | 0.831 | 0.833 | | 0.6493 - 1.0000 | | 0.571 | 0.857 | | 0.286 | | 0.952 | | | 0.286 | | 0.571 | | 0.381 | | 0.001 | | 0.415 | |  |
|  | Validation | | 0.397 | 0.716 | | 0.5581 - 0.8747 | | 0.917 | 0.353 | | 0.109 | | 0.98 | | | 0.109 | | 0.917 | | 0.195 | | 0.00000000315 | | 0.204 | |  |
| SVM | Training | | 0.955 | 0.991 | | 0.9814 - 1.0000 | | 0.967 | 0.953 | | 0.806 | | 0.993 | | | 0.806 | | 0.967 | | 0.879 | | 0.112 | | 0.879 | |  |
|  | Test | | 0.831 | 0.929 | | 0.8669 - 0.9903 | | 0.857 | 0.829 | | 0.333 | | 0.983 | | | 0.333 | | 0.857 | | 0.48 | | 0.206 | | 0.553 | |  |
|  | Validation | | 0.801 | 0.872 | | 0.7709 - 0.9725 | | 0.75 | 0.806 | | 0.25 | | 0.974 | | | 0.25 | | 0.75 | | 0.375 | | 0.2073 | | 0.402 | |  |
| KNN | Training | | 0.927 | 0.966 | | 0.9429 - 0.9900 | | 0.833 | 0.946 | | 0.758 | | 0.966 | | | 0.758 | | 0.833 | | 0.794 | | 0.4 | | 0.705 | |  |
|  | Test | | 0.818 | 0.809 | | 0.6412 - 0.9772 | | 0.429 | 0.857 | | 0.231 | | 0.937 | | | 0.231 | | 0.429 | | 0.3 | | 0.2 | | 0.36 | |  |
|  | Validation | | 0.927 | 0.702 | | 0.5281 - 0.8753 | | 0.333 | 0.978 | | 0.571 | | 0.944 | | | 0.571 | | 0.333 | | 0.421 | | 0.4 | | 0.274 | |  |
| RandomForest | Training | | 0.989 | 1 | | 0.9991 - 1.0000 | | 0.967 | 0.993 | | 0.967 | | 0.993 | | | 0.967 | | 0.967 | | 0.967 | | 0.4 | | 0.98 | |  |
|  | Test | | 0.714 | 0.735 | | 0.5438 - 0.9256 | | 0.571 | 0.729 | | 0.174 | | 0.944 | | | 0.174 | | 0.571 | | 0.267 | | 0.2 | | 0.198 | |  |
|  | Validation | | 0.854 | 0.754 | | 0.6153 - 0.8919 | | 0.417 | 0.892 | | 0.25 | | 0.947 | | | 0.25 | | 0.417 | | 0.312 | | 0.3 | | 0.22 | |  |
| ExtraTrees | Training | | 0.831 | 1 | | 1.0000 - 1.0000 | | 0 | 1 | | 0 | | 0.831 | | | 0 | | 0 | | NaN | | 1 | | 1 | |  |
|  | Test | | 0.909 | 0.779 | | 0.5407 - 1.0000 | | 0.429 | 0.957 | | 0.5 | | 0.944 | | | 0.5 | | 0.429 | | 0.462 | | 0.3 | | 0.328 | |  |
|  | Validation | | 0.748 | 0.667 | | 0.5044 - 0.8296 | | 0.417 | 0.777 | | 0.139 | | 0.939 | | | 0.139 | | 0.417 | | 0.208 | | 0.2 | | 0.145 | |  |
| XGBoost | Training | | 0.994 | 1 | | 1.0000 - 1.0000 | | 0.967 | 1 | | 1 | | 0.993 | | | 1 | | 0.967 | | 0.983 | | 0.662 | | 1 | |  |
|  | Test | | 0.429 | 0.64 | | 0.4716 - 0.8080 | | 0.857 | 0.386 | | 0.122 | | 0.964 | | | 0.122 | | 0.857 | | 0.214 | | 0.051 | | 0.232 | |  |
|  | Validation | | 0.57 | 0.695 | | 0.5750 - 0.8141 | | 0.833 | 0.547 | | 0.137 | | 0.974 | | | 0.137 | | 0.833 | | 0.235 | | 0.06056 | | 0.251 | |  |
| LightGBM | Training | | 0.933 | 0.983 | | 0.9684 - 0.9978 | | 0.967 | 0.926 | | 0.725 | | 0.993 | | | 0.725 | | 0.967 | | 0.829 | | 0.283 | | 0.823 | |  |
|  | Test | | 0.234 | 0.5 | | 0.2653 - 0.7347 | | 0.857 | 0.171 | | 0.094 | | 0.923 | | | 0.094 | | 0.857 | | 0.169 | | 0.064 | | 0.129 | |  |
|  | Validation | | 0.444 | 0.519 | | 0.4288 - 0.6090 | | 0.75 | 0.417 | | 0.1 | | 0.951 | | | 0.1 | | 0.75 | | 0.176 | | 0.06916 | | 0.207 | |  |
| GradientBoosting | Training | | 0.966 | 0.998 | | 0.9947 - 1.0000 | | 0.967 | 0.966 | | 0.853 | | 0.993 | | | 0.853 | | 0.967 | | 0.906 | | 0.314 | | 0.91 | |  |
|  | Test | | 0.494 | 0.589 | | 0.3540 - 0.8235 | | 0.714 | 0.471 | | 0.119 | | 0.943 | | | 0.119 | | 0.714 | | 0.204 | | 0.082 | | 0.183 | |  |
|  | Validation | | 0.318 | 0.579 | | 0.4342 - 0.7229 | | 0.833 | 0.273 | | 0.09 | | 0.95 | | | 0.09 | | 0.833 | | 0.163 | | 0.06653 | | 0.146 | |  |
| AdaBoost | Training | | 0.972 | 0.99 | | 0.9785 - 1.0000 | | 0.933 | 0.98 | | 0.903 | | 0.986 | | | 0.903 | | 0.933 | | 0.918 | | 0.497 | | 0.923 | |  |
|  | Test | | 0.299 | 0.524 | | 0.3312 - 0.7178 | | 0.857 | 0.243 | | 0.102 | | 0.944 | | | 0.102 | | 0.857 | | 0.182 | | 0.019 | | 0.168 | |  |
|  | Validation | | 0.464 | 0.51 | | 0.4246 - 0.5946 | | 0.917 | 0.424 | | 0.121 | | 0.983 | | | 0.121 | | 0.917 | | 0.214 | | 0.02275 | | 0.235 | |  |
| MLP | Training | | 0.91 | 0.974 | | 0.9543 - 0.9935 | | 0.933 | 0.905 | | 0.667 | | 0.985 | | | 0.667 | | 0.933 | | 0.778 | | 0.262 | | 0.763 | |  |
|  | Test | | 0.74 | 0.745 | | 0.4861 - 1.0000 | | 0.571 | 0.757 | | 0.19 | | 0.946 | | | 0.19 | | 0.571 | | 0.286 | | 0.211 | | 0.3 | |  |
|  | Validation | | 0.861 | 0.883 | | 0.7608 - 1.0000 | | 0.75 | 0.871 | | 0.333 | | 0.976 | | | 0.333 | | 0.75 | | 0.462 | | 0.2443 | | 0.49 | |  |
| Table S3 The performance of eleven machine learning models for combined thrombus. | | | | | | | | | | | | | | | | | | | | | | | |  |  |  |
|  |  |  |  |  |  |  |  |  |  |  |  |  |  |  |  |  |  |  |  |  |  |  |  |  |  |  |
| Models | Groups | Accuracy | | | AUC | 95% CI | Sensitivity | | | Specificity | | PPV | | NPV | Precision | | Recall | | F1 | | Threshold | | MCC |  |  |  |
| LR | Training | 0.938 | | | 0.993 | 0.9854 - 1.0000 | 0.967 | | | 0.932 | | 0.744 | | 0.993 | 0.744 | | 0.967 | | 0.841 | | 0.2364 | | 0.836 |  |  |  |
|  | Test | 0.753 | | | 0.927 | 0.8473 - 1.0000 | 0.857 | | | 0.743 | | 0.25 | | 0.981 | 0.25 | | 0.857 | | 0.387 | | 0.04271 | | 0.456 |  |  |  |
|  | Validation | 0.808 | | | 0.869 | 0.7776 - 0.9611 | 0.75 | | | 0.813 | | 0.257 | | 0.974 | 0.257 | | 0.75 | | 0.383 | | 0.09833 | | 0.41 |  |  |  |
| NaiveBayes | Training | 0.792 | | | 0.918 | 0.8778 - 0.9592 | 0.933 | | | 0.764 | | 0.444 | | 0.983 | 0.444 | | 0.933 | | 0.602 | | 0.02597 | | 0.57 |  |  |  |
|  | Test | 0.857 | | | 0.906 | 0.7984 - 1.0000 | 0.714 | | | 0.871 | | 0.357 | | 0.968 | 0.357 | | 0.714 | | 0.476 | | 0.00007288 | | 0.529 |  |  |  |
|  | Validation | 0.457 | | | 0.745 | 0.6075 - 0.8829 | 0.917 | | | 0.417 | | 0.12 | | 0.983 | 0.12 | | 0.917 | | 0.212 | | 1.864E-16 | | 0.232 |  |  |  |
| SVM | Training | 0.978 | | | 0.999 | 0.9964 - 1.0000 | 0.967 | | | 0.98 | | 0.906 | | 0.993 | 0.906 | | 0.967 | | 0.935 | | 0.1963 | | 0.944 |  |  |  |
|  | Test | 0.883 | | | 0.935 | 0.8785 - 0.9909 | 0.857 | | | 0.886 | | 0.429 | | 0.984 | 0.429 | | 0.857 | | 0.571 | | 0.3202 | | 0.643 |  |  |  |
|  | Validation | 0.887 | | | 0.826 | 0.6901 - 0.9621 | 0.583 | | | 0.914 | | 0.368 | | 0.962 | 0.368 | | 0.583 | | 0.452 | | 0.3556 | | 0.463 |  |  |  |
| KNN | Training | 0.888 | | | 0.96 | 0.9363 - 0.9840 | 0.467 | | | 0.973 | | 0.778 | | 0.9 | 0.778 | | 0.467 | | 0.583 | | 0.4 | | 0.751 |  |  |  |
|  | Test | 0.883 | | | 0.651 | 0.4355 - 0.8665 | 0 | | | 0.971 | | 0 | | 0.907 | 0 | | 0 | | NaN | | 0.4 | | 0.371 |  |  |  |
|  | Validation | 0.914 | | | 0.768 | 0.6133 - 0.9233 | 0.417 | | | 0.957 | | 0.455 | | 0.95 | 0.455 | | 0.417 | | 0.435 | | 0.2 | | 0.311 |  |  |  |
| RandomForest | Training | 0.989 | | | 1 | 1.0000 - 1.0000 | 0.933 | | | 1 | | 1 | | 0.987 | 1 | | 0.933 | | 0.966 | | 0.5 | | 1 |  |  |  |
|  | Test | 0.883 | | | 0.763 | 0.5932 - 0.9333 | 0.143 | | | 0.957 | | 0.25 | | 0.918 | 0.25 | | 0.143 | | 0.182 | | 0.3 | | 0.283 |  |  |  |
|  | Validation | 0.927 | | | 0.85 | 0.7282 - 0.9714 | 0.25 | | | 0.986 | | 0.6 | | 0.938 | 0.6 | | 0.25 | | 0.353 | | 0.4 | | 0.479 |  |  |  |
| ExtraTrees | Training | 0.831 | | | 1 | 1.0000 - 1.0000 | 0 | | | 1 | | 0 | | 0.831 | 0 | | 0 | | NaN | | 1 | | 1 |  |  |  |
|  | Test | 0.844 | | | 0.868 | 0.7870 - 0.9497 | 0.429 | | | 0.886 | | 0.273 | | 0.939 | 0.273 | | 0.429 | | 0.333 | | 0.3 | | 0.456 |  |  |  |
|  | Validation | 0.801 | | | 0.782 | 0.6332 - 0.9309 | 0.583 | | | 0.82 | | 0.219 | | 0.958 | 0.219 | | 0.583 | | 0.318 | | 0.2 | | 0.231 |  |  |  |
| XGBoost | Training | 0.994 | | | 1 | 1.0000 - 1.0000 | 0.967 | | | 1 | | 1 | | 0.993 | 1 | | 0.967 | | 0.983 | | 0.6885 | | 1 |  |  |  |
|  | Test | 0.636 | | | 0.808 | 0.6874 - 0.9289 | 0.857 | | | 0.614 | | 0.182 | | 0.977 | 0.182 | | 0.857 | | 0.3 | | 0.07594 | | 0.356 |  |  |  |
|  | Validation | 0.477 | | | 0.691 | 0.5521 - 0.8292 | 0.833 | | | 0.446 | | 0.115 | | 0.969 | 0.115 | | 0.833 | | 0.202 | | 0.05958 | | 0.195 |  |  |  |
| LightGBM | Training | 0.966 | | | 0.997 | 0.9924 - 1.0000 | 0.967 | | | 0.966 | | 0.853 | | 0.993 | 0.853 | | 0.967 | | 0.906 | | 0.3002 | | 0.91 |  |  |  |
|  | Test | 0.519 | | | 0.619 | 0.4607 - 0.7781 | 0.714 | | | 0.5 | | 0.125 | | 0.946 | 0.125 | | 0.714 | | 0.213 | | 0.06995 | | 0.206 |  |  |  |
|  | Validation | 0.351 | | | 0.55 | 0.4373 - 0.6628 | 0.917 | | | 0.302 | | 0.102 | | 0.977 | 0.102 | | 0.917 | | 0.183 | | 0.06338 | | 0.182 |  |  |  |
| GradientBoosting | Training | 0.989 | | | 0.998 | 0.9931 - 1.0000 | 0.933 | | | 1 | | 1 | | 0.987 | 1 | | 0.933 | | 0.966 | | 0.3349 | | 0.98 |  |  |  |
|  | Test | 0.442 | | | 0.659 | 0.4913 - 0.8271 | 0.857 | | | 0.4 | | 0.125 | | 0.966 | 0.125 | | 0.857 | | 0.218 | | 0.07898 | | 0.239 |  |  |  |
|  | Validation | 0.781 | | | 0.548 | 0.3476 - 0.7484 | 0.333 | | | 0.82 | | 0.138 | | 0.934 | 0.138 | | 0.333 | | 0.195 | | 0.1535 | | 0.154 |  |  |  |
| AdaBoost | Training | 0.961 | | | 0.996 | 0.9916 - 1.0000 | 0.967 | | | 0.959 | | 0.829 | | 0.993 | 0.829 | | 0.967 | | 0.892 | | 0.4769 | | 0.894 |  |  |  |
|  | Test | 0.714 | | | 0.619 | 0.3694 - 0.8693 | 0.571 | | | 0.729 | | 0.174 | | 0.944 | 0.174 | | 0.571 | | 0.267 | | 0.03252 | | 0.275 |  |  |  |
|  | Validation | 0.291 | | | 0.543 | 0.3977 - 0.6880 | 0.833 | | | 0.245 | | 0.087 | | 0.944 | 0.087 | | 0.833 | | 0.157 | | 0.01818 | | 0.155 |  |  |  |
| MLP | Training | 0.921 | | | 0.986 | 0.9735 - 0.9985 | 0.967 | | | 0.912 | | 0.69 | | 0.993 | 0.69 | | 0.967 | | 0.806 | | 0.2872 | | 0.798 |  |  |  |
|  | Test | 0.922 | | | 0.861 | 0.6969 - 1.0000 | 0.571 | | | 0.957 | | 0.571 | | 0.957 | 0.571 | | 0.571 | | 0.571 | | 0.2881 | | 0.633 |  |  |  |
|  | Validation | 0.815 | | | 0.741 | 0.6084 - 0.8736 | 0.5 | | | 0.842 | | 0.214 | | 0.951 | 0.214 | | 0.5 | | 0.3 | | 0.215 | | 0.292 |  |  |  |

|  | Table S4. TOAST Classifications by MCE Subgroups | | | | |
| --- | --- | --- | --- | --- | --- |
| Characteristics | | | MCE+ | MCE- |  |
| TOAST | | 1 LAA,n(%) | 12(6.45) | 174(93.55) |  |
|  | | 2 CE,n(%) | 26(25.49) | 76(74.51) |  |
|  | | 3 Other types,n(%) | 11(9.32) | 107(90.68) |  |

Note.TOAST (Trial of Org 10172 in Acute Stroke Treatment) classification:

TOAST 1 is defined as large artery atherosclerotic (LAA) stroke; TOAST 2

is defined as cardioembolic stroke; and TOAST 3 is defined as the aggregate

of small vessel occlusion (SVO), stroke of other determined etiology (SOE), and

stroke of undetermined etiology (SUE).

| Table S5. In-hospital Mortality and Etiological Distribution of Deaths Stratified by MCE Status | | | | | |  |
| --- | --- | --- | --- | --- | --- | --- |
| Subgroup | Total Cases,n | Deaths,n | Mortality Rate,n/N(%) | Causes of Death | |  |
|  |  |  |  | Neurological (n/N, %) | Non-neurological (n/N, %) |  |
| MCE+ | 49 | 29 | 29/49(59.2) | 27/29(93.1) | 2/29(6.8) |  |
|  |  |  |  |  |  |  |
| MCE- | 357 | 2 | 2/357(0.6) | 1/2(50.0) | 1/2(50.0) |  |
|  |  |  |  |  |  |  |
